# Developing tumor-specific PRO-CTCAE item sets: analysis of a cross-sectional survey in three German outpatient cancer centers

**DOI:** 10.1186/s12885-023-11115-7

**Published:** 2023-07-05

**Authors:** Maximilian Günther, Leopold Hentschel, Markus Schuler, Theresa Müller, Katharina Schütte, Yon-Dschun Ko, Ingo Schmidt-Wolf, Ulrich Jaehde

**Affiliations:** 1 Institute of Pharmacy, Department of Clinical Pharmacy, University of Bonn, An der Immenburg 4, 53121 Bonn, Germany; 2 National Center for Tumor Diseases (NCT/UCC), University Hospital Carl Gustav Carus, Dresden, Germany; 3 Clinic and Polyclinic for Internal Medicine, University Hospital Carl Gustav Carus, Technical University Dresden, Dresden, Germany; 4 Department of Internal Medicine, Johanniter Hospital, Bonn, Germany; 5 Department of Integrated Oncology, CIO Bonn, University Hospital, Bonn, Germany

**Keywords:** Patient-reported outcomes, PRO-CTCAE, Breast cancer, Symptom burden, Multiple myeloma, Prostate cancer

## Abstract

**Background:**

To include the patient perspective in the assessment of adverse events in oncology, a patient-reported outcomes (PRO) version of the Common Terminology Criteria for Adverse Events (CTCAE) was developed by the US National Cancer Institute, the so called PRO-CTCAE. The objective of this study was the development of disease-specific PRO-CTCAE item sets for patients with breast cancer (BC), multiple myeloma (MM), and prostate cancer (PC).

**Methods:**

The cross-sectional survey was conducted at three German outpatient cancer centers. Prevalence and importance of the 78 PRO-CTCAE symptoms were assessed using a patient questionnaire. To select the most relevant PRO-CTCAE items for each tumor entity, symptoms were ranked based on patient answers.

**Results:**

101 patients with BC, 107 with MM, and 66 with PC participated. The final item sets contained 21 symptoms (BC) or 19 symptoms (MM and PC), respectively. Eight symptoms (fatigue, muscle pain, insomnia, joint pain, general pain, dizziness, shortness of breath, and swelling) were represented in all three item sets. Fatigue was the symptom with the highest ranking across item sets followed by insomnia. Symptoms with the highest rankings represented in only one item set were symptoms affecting the urogenital system in the PC item set, blurred vision in the BC item set, and decreased appetite in the MM item set.

**Conclusions:**

Individual PRO-CTCAE item sets for a German patient population were developed for the three tumor entities on the basis of patients’ differences in symptom profiles and perceptions. The quality and psychometric criteria of the newly compiled item sets should be evaluated in validation studies.

## Background

In oncology, adverse events are assessed by physicians using the Common Terminology Criteria for Adverse Events (CTCAE) [1]. This physician-reported assessment often differs from the patients’ experience. In a study on patients with chronic myeloid leukemia, the severity of symptoms was underestimated by physicians most frequently for fatigue (51%), muscle cramps (49%), and musculoskeletal pain (42%), whereas the physicians overestimated the health status in 67% of case [2]. In an evaluation involving three randomized clinical trials with elderly breast cancer or advanced non-small-cell lung cancer patients, physicians underreported symptoms of any severity in 41–74% and severe symptoms in 13–50% of cases [3]. These findings were confirmed by a systematic review on 28 studies involving patients with various cancer types [4]. It concludes that the association between CTCAE and patient-reported outcomes (PRO) was poor to moderate with a large variation across studies [4].

To incorporate the patient perspective into the assessment of symptoms, Basch et al. conducted a randomized controlled trial. Patients in the intervention group underwent a weekly electronic monitoring of PRO symptoms with automated alerts to clinicians. Patients in the control group were treated with standard oncology care. Health-related quality of life after six months improved more and worsened less often in the intervention group. Patients who underwent the electronic PRO symptom monitoring were less frequently admitted to the emergency department or hospital and remained longer under therapy. Even the overall survival of the patients was prolonged [5]. Yet methodological limitations were critically discussed, these findings were affirmed by a further trial [6].

To complement the physician-based assessment of adverse events, the United States National Cancer Institute has developed a PRO version of the CTCAE [7]. Out of the 790 adverse events listed in the CTCAE, 78 were considered to be appropriate to be rated directly by the patients. Plain language terms and up to three items characterizing severity, frequency, and interference with daily activities were designed for every symptomatic adverse event and refined in a cognitive interviewing study creating a library consisting of 124 items. The items were evaluated regarding their construct validity, reliability, responsiveness, and between-mode equivalence [8, 9]. The PRO-CTCAE item library was translated into more than 30 languages including German [7]. A German PRO-CTCAE core item set containing 31 items for patients with chemotherapy was validated by our group [10]. The complementary nature of PRO-CTCAE to CTCAE is illustrated by the fact that patients´ and physicians´ answers to questions regarding complementary symptoms of PRO-CTCAE and CTCAE differ a lot. The agreement was poor and patients tended to grade symptoms more severe than physicians [11].

The disease symptoms and treatment options differ considerably among tumor entities. Therefore, individual symptoms are not equally relevant to all cancer patients. In order to maximize the informative value of the PRO-CTCAE questionnaire and simultaneously minimize patient burden, it seems prudent to include only a selection of items with the highest impact on the patients in a PRO-CTCAE questionnaire. In consequence, tumor entity-specific PRO-CTCAE item sets for patients with lung cancer, bladder cancer, hepatocellular cancer, melanoma receiving immunotherapy, and prostate cancer have been developed [12–16].

The aim of this project was to identify the most relevant PRO-CTCAE symptoms and to develop item sets for patients with different tumor diseases receiving active anticancer drug therapy. Our selection of tumor entities intended to cover solid, hematological, gender-specific, and frequently occurring malignancies. Based on these criteria, the following three tumor entities were identified for this project: breast cancer, multiple myeloma, and prostate cancer.

## Methods

### Study design and participants

This project was a multicentric, cross-sectional, and non-interventional patient survey. It was conducted at the Center for Integrated Oncology (CIO) of the University Hospital in Bonn, the Johanniter Hospital in Bonn, and the National Center for Tumor Diseases (NCT/UCC) at the University Hospital in Dresden. Beside the patients´ assessments of the prevalence and importance of therapy-associated symptoms contained in the PRO-CTCAE item library, the underlying tumor medication and disease-specific data were recorded.

The study was approved by the Ethics Committee of the Medical Faculty of the University of Bonn (Lfd. Nr. 405/17) and by the relevant institutions of each participating center. It was carried out in accordance with the applicable German and European legal provisions and it was performed in accordance with the principles of the Declaration of Helsinki.

Cancer patients with breast cancer, prostate cancer, and multiple myeloma matched the inclusion criteria, if they were at least 18 years old and underwent active anticancer drug treatment of their disease. Patients with insufficient knowledge of the German language were excluded because all relevant documents and information were in German. A prespecified sample of 100 patients for every tumor entity should be included consecutively in the study. This sample size is based on the assumption of an acceptable error margin of 10% and a 95% confidence level.

For recruitment, patients matching the inclusion criteria were identified by the treating physicians at the study centers and invited to participate. Patients were informed orally and in writing about the nature, significance, and scope of the patient survey and signed a written informed consent. Patients were recruited between February 2018 and April 2019 at the Center for Integrated Oncology (CIO) of the University Hospital in Bonn, the Johanniter Hospital Bonn, and the National Center for Tumor Diseases (NCT/UCC) in Dresden.

For further data processing and statistical analysis of the anonymized data, Microsoft® Access® 2019 (Microsoft Corporation, Redmond, USA), Microsoft® Excel® 2019 (Microsoft Corporation, Redmond, USA), and IBM® SPSS® Statistics Version 27.0 for Windows (IBM Corporation, Armonk, USA) were used.

Descriptive statistics were performed for patient characteristics and medication data. As applicable, mean values with standard deviations (SD) or the median with interquartile range (IQR) were calculated. Frequencies were described as absolute number and percentage. For inductive statistics in the item-redundancy analysis, a p-value of < 0.05 was considered as statistically significant.

### Patient questionnaire

The EORTC guidelines for developing questionnaire modules include four phases of the development process: (1) Generation of relevant PRO issues, (2) Converting the PRO issues into an item set, (3) Pre-testing of the item set, and (4) Large-scale international field testing [17].

While developing the PRO-CTCAE item library by the US National Cancer Institute, the above-mentioned steps have already been taken for the item library in general. From the CTCAE catalogue containing 790 adverse events, 78 symptomatic adverse events that are relevant for cancer patients in general, were derived (Phase 1). Plain language terms and up to three items were designed for every symptomatic adverse event and refined in a cognitive interviewing study (Phase 2). The items were evaluated regarding their construct validity, reliability, responsiveness, and between-mode equivalence (Phase 3 and 4) [8, 9, 18].

Despite being a valid PRO instrument in general, the complete PRO-CTCAE question pool with 124 items is too extensive to be administered in total. This circumstance raises the question which symptoms are relevant to patients with different cancer types. The question refers to phase 1 of the development process in which the foundation for high content validity of the PRO tool is laid. For compiling the relevant PRO issues, three sources should be used: literature, patients, and healthcare professionals. PRO-CTCAE is a well-characterized instrument for detecting symptomatic adverse events, PRO-CTCAE terms are already based on literature review [8], and it is known that the physician-reported assessment often differs from the patients’ experience [2–4]. Therefore, the patient perspective was chosen over the perspective of healthcare professionals. This is also encouraged by the EORTC guidelines [17]. Consequently, a questionnaire-based patient survey on the prevalence and importance of the symptoms was conducted.

In order to minimize the time burden for patients, a newly developed questionnaire was used in this project consisting of two parts.

**Fig. 1:**
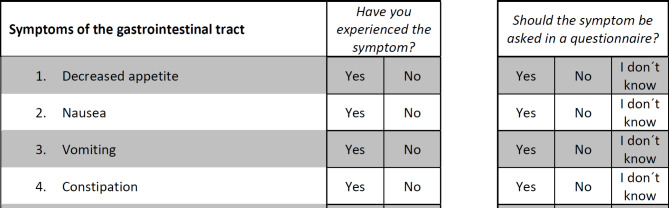
Example questions from the patient questionnaire (English translation)

In the first part, patients were asked to answer if the 78 symptoms have ever occurred during their tumor therapy and if they think that the symptoms should be asked for in a questionnaire for patients suffering from their tumor disease. In this study, the symptoms “Nail ridging” and “Nail discoloration” were condensed to one item (“Nail ridging or discoloration”), resulting in 77 questions in the patient questionnaire. The questions about the prevalence of the symptoms could be answered with “Yes” or “No”. The questions about the importance of the symptoms could be answered with “Yes”, “No”, or “I don’t know”. This procedure was adapted from the Guidelines for Developing Questionnaire Modules of the EORTC Quality of Life Group [17]. An example for how the questions in the administered questionnaire looked like is given in Fig. 1.

The second part of the questionnaire contained questions on sociodemographic data, the characteristics of tumor disease, and tumor therapy, which are essential for the characterization of the study populations. Additional information, that could not be obtained from the patients themselves, on disease-specific characteristics, cancer treatment modalities, tumor medication, and supportive care medication for 14 indications was collected retrospectively from patient records using case report forms that were filled in by health care professionals at the study centers.

### Item selection

To build the entity-specific PRO-CTCAE item sets, the most prevalent and most important symptoms of the patient questionnaires needed to be identified. An approach based on the clinical impact method (CIM) was used for the item selection. The CIM focuses on the severity and importance of items rated by the patients and is applicable for small sample sizes [19]. In this study, the CIM was modified. Instead of the symptom severity, the symptom prevalence was used.

### Symptom rating and ranking

According to the CIM, patients rate the severity and the importance of the questionnaire items. The ratings are combined to the clinical impact (CI) by adding the mean importance rating per item (I) and the mean severity rating per item (S) using Eq. 1 [19].

1 $$\text{C}\text{I}=\text{I}+\text{S}$$

I: Symptom importance

S: Symptom severity

As described above, the symptom prevalence was used in this study instead of severity because severity is only one possible attribute of the PRO-CTCAE symptoms and not included in all symptom scales. In addition, the occurrence of a symptom may be assessed more reliably than its severity if the symptom has appeared in the past.

The scales were scored with “Yes” = 1 and “No” = 0 for the symptom prevalence and with “Yes” = 1, “No” = 0, and “I don’t know” = 0.5 for the symptom importance. In the first step, two separate scores for the symptom prevalence and the symptom importance were calculated. Therefore, the values for every symptom were added and divided by the number of completed questionnaires to avoid distortions due to missing values. By doing so, the values of the scores displayed values between 0 and 1. The values were ranked from 1 (symptom with highest score) to 77 (symptom with lowest score). In the second step, the rankings for the symptom prevalence (1 to 77) and the symptom importance (1 to 77) were added to the combined prevalence-importance (P-I) score. According to their combined P-I score, the symptoms were then ranked from lowest to highest. The lower the combined score the higher were prevalence and importance.

Finally, the PRO-CTCAE items related to the symptoms with the lowest combined P-I scores were included. For every PRO-CTCAE symptom, up to three items are available. The items of one symptom were not split in the selection process. The goal of item selection was to reduce the number of items for the entity-specific PRO-CTCAE item sets to a maximum of 40. The EORTC QLQ-C30 (30 items) plus entity-specific modules like QLQ-MY20 (multiple myeloma, 20 items), QLQ-BR23 (breast cancer, 23 items) or QLQ-PR25 (prostate cancer, 25 items) contain about 50 items in total [20]. The Functional Assessment of Cancer Therapy (FACT) questionnaires of the FACIT group consist of 27 items for the FACT-G (general) questionnaire, 37 items for the FACT-B (breast cancer), 41 items for the FACT-MM (multiple myeloma) and 39 items for FACT-P (prostate cancer) [21]. Whereas these instruments cover a broad range of domains of health-related quality of life, PRO-CTCAE focuses on symptomatic adverse events only. In general, it is recommended that the answering of a PRO questionnaire should be limited to 10 to 15 minutes or less if the questionnaire is administered repeatedly to minimize the response burden for patients [22]. Answering a 28-item PRO-CTCAE questionnaire takes four to six minutes [8]. Therefore, a number of 40 items was chosen as a cut-off because it is considered as a not too burdensome number of questions.

### Item redundancy analysis

The symptoms contained in each item set were investigated for inter-item correlations to reveal redundancies among the selected symptoms.

To investigate the association between the symptom items, the φ coefficient for dichotomous variables by Pearson was used. The φ coefficient can take values between − 1 and + 1. Values around 0 indicate weak correlation. Values of ± 1 indicate perfect positive or negative correlation. Values of ± 0.8 or higher indicate strong correlation between two variables [23].

The response options for the questions on symptom prevalence (“Yes” and “No”) are dichotomous already. The response scale for the questions on symptom importance (“Yes”, “No”, and “I don’t know”) were translated to the dichotomous answers “Yes” and “not Yes” (including “No” and “I don’t know”) in the course of the item-redundancy analysis.

Pairs of symptom items with a ϕ value of ≥ ± 0.8 reveal possible redundancies. To analyze the significance of these correlations and to uncover random correlations, Fisher´s exact test was conducted [24, 25].

## Results

### Study population

The sociodemographic and disease-related characteristics of the study populations are shown in Table 1.

**Table 1:** Sociodemographic and disease-related characteristics of the study populations [n (%)]

	Breast cancer	Multiple myeloma	Prostate cancer
**Number of patients**	**101**	**107**	**66**
**Study center**			
CIO Bonn Johanniter Hospital Bonn University Hospital Dresden	80 (79.2) 21 (20.8) 0 (0)	35 (32.7) 12 (11.2) 60 (56.1)	6 (9.1) 0 (0) 60 (90.9)
**Gender**			
Male Female	0 (0) 101 (100)	67 (62.6) 40 (37.4)	66 (100) 0 (0)
**Age**			
Median [years]	58 (IQR: 16, range: 28–84, mean 58.0, SD 11.4)	62 (IQR: 13, range: 33–83, mean: 62.0, SD: 9.1)	76 (IQR: 8, range: 59–94, mean: 74.5, SD: 6.9)
**Time since the first diagnosis of cancer**			
Median [months]	14 (IQR: 67, range: 1–506, mean: 59, SD: 86.9)	59 (IQR: 69, range: 2–255, mean: 63.7, SD: 52.8)	27 (IQR: 60, range: 1–190, mean: 41.7, SD: 43.3)
**Current therapy situation**			
Outpatient therapy Inpatient therapy	101 (100) 0 (0)	87 (81.3) 20 (18.7)	56 (84.9) 10 (15.1)
**Current therapy intention**			
Curative Palliative Other/unknown	32 (31.7) 53 (52.5) 16 (15.8)	35 (32.7) 68 (63.6) 4 (3.7)	24 (36.4) 30 (45.5) 12 (18.2)
**Current therapy sequence**			
Adjuvant Neoadjuvant Other/unknown	52 (51.5) 23 (22.8) 26 (25.7)	n.a. n.a. n.a.	3 (4.5) 0 (0) 63 (95.5)
Current additional therapy modalities*			
Radiation Surgery No additional therapy Other/unknown	21 (20.8) 20 (19.8) 60 (59.4) 0 (0)	3 (2.8) 0 (0) 90 (84.1) 14 (13.1)#	29 (43.9) 1 (1.5) 27 (41) 9 (13.6)
**Metastases**			
Patients with metastases Number of metastases per patient with metastases	54 (53.5) 1.7 (SD: 0.9, median: 1, IQR: 1, range: 1–4)	n.a. n.a.	20 (30.3) 1.1 (SD: 0.2, median: 1, IQR: 0, range: 1–2)
**Relapses**			
Patients with relapse	22 (21.8)	27 (25.2)	18 (27.3)

n.a. = not applicable, IQR = interquartile range, SD = standard deviation, * = besides drug therapy, # patients with autologous stem cell transplantation

The time since the first diagnosis of cancer for patients of all three tumor entities is quite long (breast cancer: 14 months [IQR: 67, range: 1–506, mean: 59, SD: 86.9]; multiple myeloma: 59 months [IQR: 69, range: 2–255, mean: 63.7, SD: 52.8]; prostate cancer: 27 months [IQR: 60, range: 1–190, mean: 41.7, SD: 43.3]). Consequently, the long time period between diagnosis and completing the survey may have led to a recall bias.

53.5% (n = 54) of the breast cancer patients had metastases. The most often occurring metastases were located in bones (n = 34), liver (n = 20), and lung (n = 15). 22 patients (21.8%) had a relapse of their disease. 15 of them had only one relapse. An estrogen receptor-positive disease occurred in 65 patients (64.4%). Progesterone receptor-positive were 46 patients (45.6%) and HER2-positive 44 patients (43.6%).

25.2% (n = 27) of the multiple myeloma patients had a relapse of their disease. 22 of them had only one relapse. 14 patients (13.1%) were currently undergoing autologous stem cell transplantation (SCT). 77 patients (72.0%) received at least one autologous SCT during former therapy lines.

30.3% (n = 20) of the prostate cancer patients had metastases. With one exception the metastases were located in bones (n = 20). 18 patients (27.3%) had a relapse of their disease. Most patients belonged to the high-risk Gleason grade group 5 (n = 26, 39.4%).

**Fig. 2:**
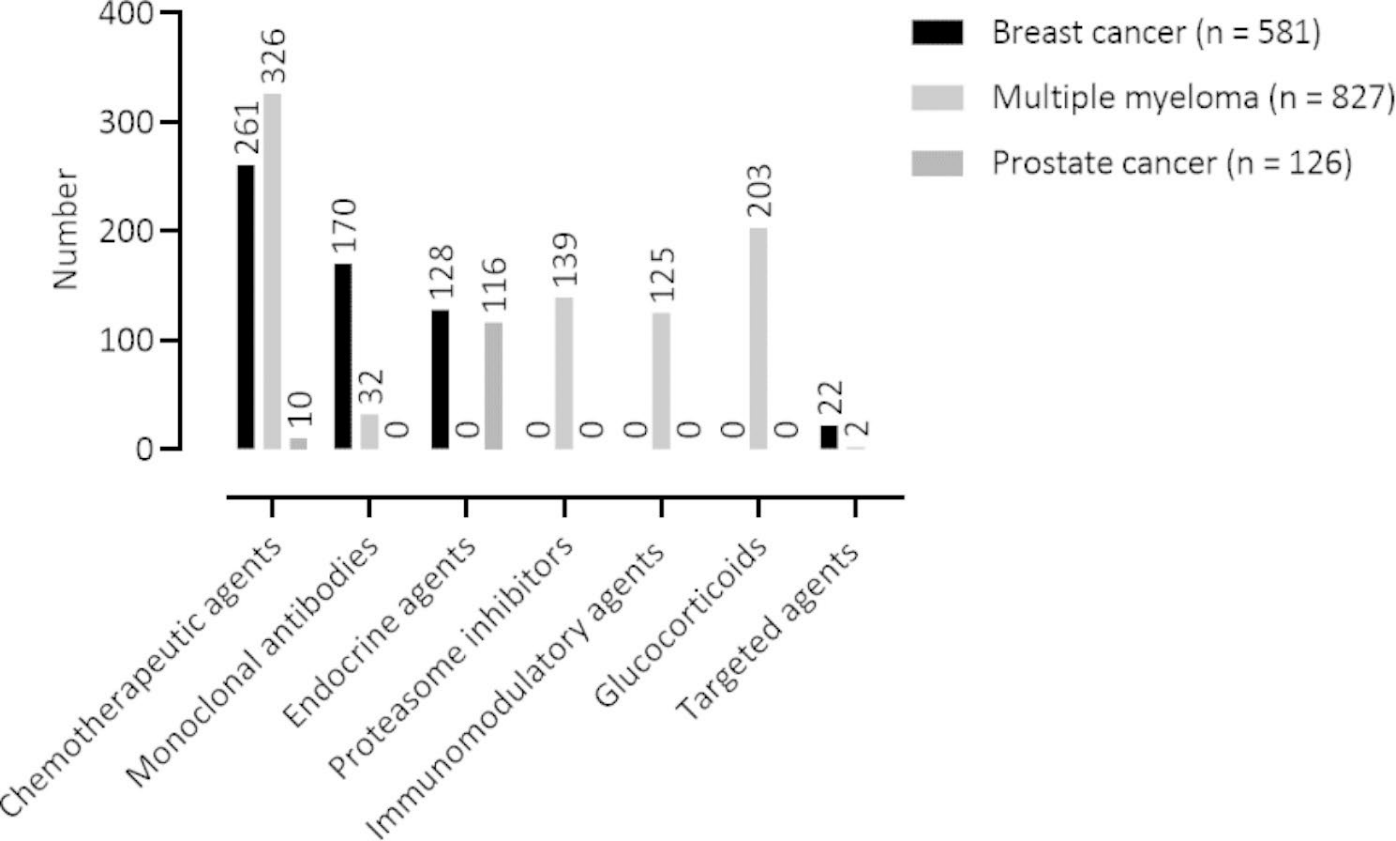
Drug classes used in the therapy of the participating breast cancer (n = 101), multiple myeloma (n = 107), and prostate cancer (n = 66) patients

The drug classes used for the treatment of the three tumor entities are shown in Fig. 2. Overall, the mean number of anticancer drugs per patient was 7.7 (SD: 4.1, median: 7, IQR: 5, range 0–24) in the multiple myeloma patients, followed by the breast cancer patients with a mean of 5.8 (SD: 3.4, median: 5, IQR: 4, range: 0–17). The prostate cancer patients received only a mean of 1.9 (SD: 1.6, median: 2, IQR: 1, range 0–7) drugs per patient.

The most used anticancer drugs in breast cancer patients were trastuzumab (n = 91/581, 15.7%), cyclophosphamide (n = 69/581, 11.9%), epirubicin (n = 68/581, 11.7%), pertuzumab (n = 55/581, 9.5%), and docetaxel (n = 44/581, 7.6%). The most used drugs in multiple myeloma patients were dexamethasone (n = 199/827, 24.1%), bortezomib (n = 124/827, 15.0%), cyclophosphamide (n = 113/827, 13.7%), melphalan (n = 111/827, 13.4%), and lenalidomide (n = 110/827, 13.3%). The most used drugs in prostate cancer patients were not further specified LH-RH analogs (n = 32/126, 25.4%), bicalutamide (n = 24/126, 19.1%), leuprorelin (n = 23/126, 18.3%), degarelix (n = 12/126, 9.5%), and abiraterone acetate (n = 10/126, 7.9%).

Breast cancer patients were treated for a mean of 1.7 indications for supportive care medication (SD: 1.7, median: 1, IQR: 1, range: 0–6). They received most frequently medication for nausea and emesis (n = 41, 24.3%), bone complications (n = 40, 23.7%), and gastric ulcer prophylaxis (n = 18, 10.7%). Multiple myeloma patients were treated also for a mean of 1.7 supportive care medication indications (SD: 1.5, median: 1, IQR: 2, range: 0–6), most frequently for bone complications (n = 48, 26.5%), gastric ulcer prophylaxis (n = 43, 23.8%), and pain (n = 34, 18.8%). Prostate cancer patients were only treated for a mean of 0.4 supportive care medication indications (SD: 0.9, median: 0, IQR: 0, range: 0–4), the most frequently for bone complications (n = 7, 25.9%), gastric ulcer prophylaxis (n = 6, 22.2%), and pain (n = 5, 18.5%).

### Completion of questionnaires

Missing values occurred if patients did not answer a question at all or not clear enough by marking an answer on the paper-based questionnaires. Within the 101 questionnaires filled in by the breast cancer patients, the questions regarding the prevalence of the symptoms were not answered in 2.2% of cases (mean number of cases 2.2, SD 3.17, range 0–16). The questions about the importance of the symptoms were not answered in 4.2% of cases (mean number of cases 4.2, SD 1.52, range 1–10). For the 107 questionnaires of the multiple myeloma patients, the missing values amount to 0.8% of cases (mean number of cases 0.9, SD 1.38, range 0–6) for the prevalence questions and 1.6% of cases (mean number of cases 1.7, SD 1.29, range 0–6) for the importance questions. For the 66 questionnaires of the prostate cancer patients, the missing values amount to 0.2% of cases (mean number of cases 0.1, SD 0.31, range 0–1) for the prevalence questions and 5.6% of cases (mean number of cases 3.7, SD 1.77, range 1–7) for the importance questions.

### Symptom rating

#### Breast cancer

The 101 participating breast cancer patients experienced a median of 27 symptoms each (IRQ 31, range 4–53). The symptom that occurred most often was hair loss (Number 24) in 86 patients. A median of 46 symptoms per patient (IQR 36, range 0–75) was rated important.

#### Multiple myeloma

The 107 participating multiple myeloma patients experienced a median of 28 symptoms each (IRQ 14, range 7–61). The symptom that occurred most often was fatigue (Number 52) in 92 patients. A median of 48 symptoms per patient (IQR 32, range 0–75) was rated important.

#### Prostate cancer

The 66 participating prostate cancer patients experienced a median of 20 symptoms each (IRQ 13, range 4–51). The symptom that occurred most often was achieve and maintain erection (Number 64) in 59 patients. A median of 46 symptoms per patient (IQR 26, range 0–72) was rated important.

### Symptom ranking and item set compilation

The final PRO-CTCAE item set for breast cancer included 39 items related to 21 symptoms, for multiple myeloma 39 items related to 19 symptoms, and for prostate cancer 40 items related to 19 symptoms, respectively. The three tumor disease-specific PRO-CTCAE item sets ranked by their combined P-I scores are shown in Tables 2, 3 and 4.

**Table 2:** PRO-CTCAE breast cancer item set including 39 items for 21 symptoms

PRO-CTCAE term	Symptom Number	Combined P-I score	Attributes
Fatigue	52	3	S, I
Numbness and tingling	37	9	S, I
Nausea	11	16	F, S
Muscle pain	48	19	F, S, I
Insomnia	51	23	S, I
Hair loss	24	24	P
Joint pain	49	24	F, S, I
Blurred vision	41	27	S, I
Concentration	40	28	S, I
General pain	46	30	F, S, I
Diarrhea	14	33	F
Constipation	13	34	S
Taste changes	18	35	S
Dizziness	38	36	S, I
Shortness of breath	7	37	S, I
Heart palpitations	36	44	F, S
Memory	39	44	S, I
Swelling	35	45	F, S, I
Rash	21	46	P
Nail ridging*	29	47	P
Nail discoloration*	29	47	P

* Symptoms were combined in the patient questionnaire

*F: frequency; S: severity; I: interference; P: presence/absence/amount*

**Table 3:** PRO-CTCAE multiple myeloma item set including 39 items for 19 symptoms

PRO-CTCAE term	Symptom Number	Combined P-I score	Attributes
Fatigue	52	4	S, I
Numbness and tingling	37	6	S, I
General pain	46	11	F, S, I
Insomnia	51	12	S, I
Nausea	11	15	F, S
Shortness of breath	7	19	S, I
Hair loss	24	20	P
Joint pain	49	21	F, S, I
Diarrhea	14	24	F
Muscle pain	48	25	F, S, I
Anxious	50	25	F, S, I
Decreased appetite	10	30	S, I
Swelling	35	37	F, S, I
Dizziness	38	37	S, I
Heart palpitations	36	38	F, S
Concentration	40	39	S, I
Constipation	13	43	S
Taste changes	18	47	S
Mouth/throat sores	1	49	S, I

*F: frequency; S: severity; I: interference; P: presence/absence/amount*

**Table 4:** PRO-CTCAE prostate cancer item set including 40 items for 19 symptoms

PRO-CTCAE term	Symptom Number	Combined P-I score	Attributes
Insomnia	51	9	S, I
Urinary frequency	57	9	F, I
Achieve and maintain erection	64	9	S
Urinary incontinence	59	10	F, I
Ejaculation	65	10	F
Fatigue	52	11	S, I
Urinary urgency	56	12	F, I
Unable to have orgasm	61	16	P, P
Joint pain	49	21	F, S, I
Anxious	50	19	F, S, I
Decreased libido	60	21	S
General pain	46	27	F, S, I
Hot flashes	62	28	F, S
Swelling	35	32	F, S, I
Shortness of breath	7	37	S, I
Sad	53	37	F, S, I
Muscle pain	48	40	F, S, I
Painful urination	55	41	S
Dizziness	38	45	S, I

*F: frequency; S: severity; I: interference; P: presence/absence/amount*

The eight symptoms fatigue, muscle pain, insomnia, joint pain, general pain, dizziness, shortness of breath, and swelling are included in all three item sets. The symptoms with the highest rankings across the item sets were fatigue and insomnia. Symptoms with the highest rankings included in only one item set were symptoms affecting the urogenital system in the prostate cancer item set, blurred vision in the breast cancer item set, and decreased appetite in the multiple myeloma item set.

### Item redundancy analysis

For the prevalence of symptoms, high correlations with a φ coefficient of 0.8 or higher did not appear throughout the three tumor entities. Table 5 shows the φ coefficients for the correlations of the symptom prevalence.

**Table 5:** Symptom prevalence in breast cancer (n = 101), multiple myeloma (n = 107), and prostate cancer patients (n = 66), correlations with a φ coefficient ≥ 0.5

Symptoms	coefficient	Fisher´s exact test
**Breast cancer**		
General pain (46) and Joint pain (49)	0.50	0.000*
Muscle pain (48) and Joint pain (49) General pain (46) and Muscle pain (48)	0.52 0.50	0.000* 0.000*
**Multiple myeloma**		
Muscle pain (48) and Joint pain (49)	0.51	0.000*
**Prostate cancer**		
Decreased libido (60) and Delayed orgasm (61)	0.52	0.000*
Decreased libido (60) and Achieve and maintain erection (64)	0.51	0.001*
Achieve and maintain erection (64) and Ejaculation (65)	0.69	0.000*

* p < 0.05: statistically significant

For the importance of symptoms, high correlations with a φ coefficient of 0.8 or higher were observed (see Table 6). Compared to the other tumor entities, prostate cancer symptom importance shows the greatest number of high φ values. Three symptom clusters with possibly redundant symptom pairs were detected for which redundancy is pathophysiologically highly plausible: sexual, hormonally-related, and urogenital symptoms.

**Table 6:** Symptom importance in breast cancer (n = 101), multiple myeloma (n = 107), and prostate cancer patients (n = 66), correlations with a φ coefficient ≥ 0.8

Symptoms	φ coefficient	Fisher´s exact test
**Breast cancer**		
Memory (39) and Concentration (40)	0.82	0.000*
Muscle pain (48) and Joint pain (49)	0.84	0.000*
**Multiple myeloma**		
Swelling (35) and Heart palpitations (36)	0.82	0.000*
**Prostate cancer**		
General pain (46) and Joint pain (49)	0.92	0.000*
Muscle pain (48) and Joint pain (49)	0.85	0.000*
Anxious (50) and Fatigue (52)	0.82	0.000*
Painful urination (55) and Urinary urgency (56)	0.83	0.000*
Painful urination (55) and Urinary frequency (57)	0.83	0.000*
Painful urination (55) and Urinary incontinence (59)	0.83	0.000*
Urinary urgency (56) and Urinary incontinence (59)	1.00	0.000*
Decreased libido (60) and Delayed orgasm (61)	1.00	0.000*
Decreased libido (60) and Achieve and maintain erection (64)	0.88	0.000*
Decreased libido (60) and Ejaculation (65)	0.88	0.000*
Delayed orgasm (61) and Achieve and maintain erection (64)	0.88	0.000*
Delayed orgasm (61) and Ejaculation (65)	0.88	0.000*
Achieve and maintain erection (64) and Ejaculation (65)	1.00	0.000*

* p < 0.05: statistically significant

The item redundancy analysis in this study refers to prevalence and importance of the symptoms surveyed in the study’s patient questionnaire and not the attributes severity, frequency, and interference with daily activities of PRO-CTCAE. Therefore, the analysis only reveals which symptoms may be redundant within one PRO-CTCAE item set and no items were excluded from the PRO-CTCAE item sets at this stage.

## Discussion

The aim of this project was to develop PRO-CTCAE item sets for three different cancer types.

To pursue this aim, we decided to conduct a patient survey with the 78 PRO-CTCAE symptoms without further preselection. Following this, we did not reduce the number of surveyed symptoms by a literature review. This decision is based on the circumstance that data from clinical trials, that are the basis for drug approvals and summaries of product characteristics (SmPCs), are lacking PRO data [26, 27] and may therefore be affected by differences in the perception of symptoms by physicians and patients. Because cognitive interviews were already conducted to refine the PRO-CTCAE symptom terms [8] and a validated German translation already existed [28], we decided for a questionnaire-based approach. Nevertheless, a physician-based approach could be an adequate comprehensive method for further research.

In literature, various methodological approaches were conducted in order to develop disease-specific PRO-CTCAE item sets. The PRO-CTCAE item set for lung cancer and the preexisting prostate cancer item set were developed using an approach based on a preselection by literature review, interviews with patients and health care professionals, and an expert panel [12, 16]. The item set for hepatocellular cancer was based on a similar approach as well [14]. Whereas, the item sets for patients with melanoma receiving immunotherapy and bladder cancer were based on literature and SmPCs, chart audits, and patient interviews [13, 15].

Comparing the three tumor entity-specific PRO-CTCAE item sets, some symptoms appear in all three item sets. This is not surprising since fatigue as a multifactorial syndrome is the most frequently observed symptom across most malignant diseases, which is also the case for mood-related symptoms like insomnia and pain-related symptoms [29]. In particular, the breast cancer and multiple myeloma PRO-CTCAE item set have 16 symptoms in common. Nevertheless, both item sets differ from the German PRO-CTCAE core item set that was designed and validated for patients under chemotherapy [10]. Of the 16 symptoms included in the PRO-CTCAE core item set, nine are included in the breast cancer item set and 12 are included in the multiple myeloma item set. The PRO-CTCAE prostate cancer set shares six symptoms with the core item set.

Moreover, there are large differences in scope among the already published tumor disease-specific item sets. The item set for lung cancer includes 17 items for eight PRO-CTCAE symptoms, the item set for patients with melanoma receiving immunotherapy 56 items for 28 PRO-CTCAE symptoms, and the item set for bladder cancer 30 items for 15 PRO-CTCAE symptoms [12, 13, 15]. Although our newly developed and the existing PRO-CTCAE item sets are disease-specific, symptoms like fatigue and pain-related symptoms are found throughout the item sets. The contained items for each item set show that there is no clear rationale on the length of a PRO-CTCAE questionnaire. Our study adds to the existing research that ranking symptoms by their clinical impact and including them in a questionnaire using a top-down selection process from most relevant to least relevant symptom may ensure that the most relevant symptoms for patients are represented. Nevertheless, defining a reasonable cut-off value for the number of items remains challenging, because the number is not only related to the maximum tolerable questions for patients, but also to the fact that the number of relevant symptoms can differ between tumor entities. Because there is already a preexisting PRO-CTCAE item set available for prostate cancer, especially the comparison of this item set with ours is interesting. The item set by Feldmann et al. consists of 13 core outcome PRO-CTCAE symptoms (ability to achieve and maintain erection, decreased libido, inability to reach orgasm, urinary frequency, urinary urgency, urinary incontinence, painful urination, fecal incontinence, fatigue, hot flashes, feeling discouraged, sadness, and concentration) [16]. Of these, our PRO-CTCAE item sets shares 10 symptoms. Only fecal incontinence, concentration, and feeling discouraged are not included. Instead of feeling discouraged our item set includes the closely related symptom feeling anxious. This suggests that both approaches could identify the most relevant symptoms for prostate cancer patients on a reasonable accuracy. Feldmann et al. also defined PRO-CTCAE symptoms that are not included in their core outcomes but are suggested to complement the subset (hormone therapy: dry mouth; radiotherapy: bloating, sleeplessness, shortness of breath, and increased sweating; radical prostatectomy: bloating, decreased appetite, constipation) [16]. Of these symptoms, sleeplessness/insomnia and shortness of breath are also included in our item set. Unique symptoms of our item set are mainly pain-related symptoms. Including these symptoms seems to be reasonable, because bone pain is a frequently occurring symptom in patients with metastatic prostate cancer. A valuable addition in the study of Feldmann et al. is that they elaborated symptoms that are not included in the PRO-CTCAE library but relevant for prostate cancer patients [16]. This supports the recommendation, that independently of the used PRO-CTCAE item set patients should be asked for further symptoms in clinical practice.

The item redundancy analysis for the prevalence and importance of the symptoms revealed some possible redundancies. High inter-item correlations that could indicate a redundancy of symptoms appeared more often in relation to the importance of the symptoms compared to their prevalence. This difference may be explained by the fact that the question on importance also applies to symptoms that do not likely occur during the patients’ therapy but were nevertheless important according to their perception.

Especially pathophysiologically plausible symptom pairs that showed possible redundancies for prevalence and importance, like the pain-related symptoms of the breast cancer patients and the cluster of sexual and hormonally-related symptoms of the prostate cancer patients, might be candidates for exclusion from the PRO-CTCAE item sets. The final decision might be taken in a validation study of the item sets determining their psychometric quality criteria reliability and construct validity, as it was conducted for the PRO-CTCAE core item set [10]. In terms of measuring the reliability of a questionnaire, internal consistency can be determined using Cronbach´s alpha. To evaluate which items negatively influence the reliability of a scale, Alpha-if-item-deleted values can be calculated.

A strength of this study is that a reasonable number of patients from different centers could be included and the completion rate of the questionnaires was high. This ensures that the results reflect the patient perspective. However, the patient survey was only conducted in Germany and the results therefore may not be generalizable to other populations. Another limitation is that included patients were diagnosed with cancer several years ago, which can lead to recall bias regarding the perception of symptoms from the past.

The tumor disease-specific PRO-CTCAE item sets for breast cancer, multiple myeloma, and prostate cancer were designed to be valid for all patients of the respective tumor entity. Patients are treated in very different ways according to their stages of disease and clinical situation, leading to the importance of developing PRO-CTCAE item sets for different therapy situations within one tumor entity. In the future new therapy options can change the symptom profiles of the cancer diseases. Therefore, the patient survey should be repeated if significant changes in treatment are introduced to maintain validity of the item sets.

## Conclusions

Based on patient-reported differences in symptom profiles and perception, specific PRO-CTCAE item sets for a German outpatient population were developed for breast cancer, multiple myeloma, and prostate cancer. In order to validate the quality and psychometric criteria of the new item sets further studies are required.

## Data Availability

The datasets generated during and/or analyzed during the current study are available from the corresponding author on reasonable request.
